# Work, Care, and Cognition: How Job Disruptions Shape ADRD Risk Across Race, Ethnicity, and Gender

**DOI:** 10.1093/geroni/igaf122.589

**Published:** 2025-12-31

**Authors:** Barbara Mendez Campos, Tsai-Chin Cho, Wenhua Lai, Jacqui Smith, HwaJung Choi

**Affiliations:** University of Tennessee Knoxville, Knoxville, Tennessee, United States; University of Michigan School of Public Health, Ann Arbor, Michigan, United States; Wayne State University, Detroit, Michigan, United States; University of Michigan, Ann Arbor, Michigan, United States; University of Michigan, Ann Arbor, Michigan, United States

## Abstract

Continuous paid work supports cognitive health, but employment disruptions, especially among historically marginalized groups, may increase the risk of cognitive decline and Alzheimer’s disease and related dementias (ADRD). While prior research has explored employment trajectories and cognitive aging, limited evidence examines how caregiving-related employment disruptions affect cognitive outcomes, especially across race/ethnicity and gender. This study investigates the association between caregiving-related job disruptions and cognitive function among older adults, with a focus on disparities. Using data from 7,769 U.S. adults aged 55 and older in the 2018 Health and Retirement Study (HRS) and Life History Mail Survey (LHMS), we classified employment histories into caregiving-related disruptions, other disruptions, or continuous employment. Sequence analysis identified employment patterns, and multivariable linear regression models assessed the impact of caregiving-related job disruptions on cognitive function, adjusting for demographic and socioeconomic covariates. Results show that women experienced significantly more caregiving-related job disruptions than men (0.54 vs. 0.04 years, p < 0.001), and Hispanic individuals had more disruptions than non-Hispanic Whites (0.83 vs. 0.26 years, p = 0.010). Caregiving-related job disruptions were significantly associated with lower cognitive scores in both females (β=-0.07, p = 0.002) and males (β=-0.14, p = 0.03), with notable disparities among non-Hispanic White and non-Hispanic Black male caregivers. Findings suggest that caregiving-related employment disruptions may contribute to cognitive decline, particularly among non-Hispanic White males and non-Hispanic Black caregivers. Targeted policies and interventions addressing these disparities are essential to reduce ADRD risk across diverse caregiving populations.

